# Assessment of Microstructure and Release of Fluoride Ions from Selected Fissure Sealants: An In Vitro Study

**DOI:** 10.3390/ma14174936

**Published:** 2021-08-30

**Authors:** Katarzyna Fita, Maciej Dobrzyński, Marta Ziętek, Dorota Diakowska, Adam Watras, Rafal Jakub Wiglusz

**Affiliations:** 1 Department of Pediatric Dentistry and Preclinical Dentistry, Wroclaw Medical University, Krakowska 26, 50-425 Wroclaw, Poland; katarzyna.fita@umed.wroc.pl (K.F.); marta.zietek@umed.wroc.pl (M.Z.); 2 Department of Nervous System Diseases, Wroclaw Medical University, Bartla 5, 51-618 Wroclaw, Poland; dorota.diakowska@umed.wroc.pl; 3 Institute of Low Temperature and Structure Research, Polish Academy of Sciences, Okolna 2, 50-422 Wroclaw, Poland

**Keywords:** fluoride, fissure sealants, prevention, tooth decay

## Abstract

The aim of this study was to compare fluoride release from four selected fissure sealants: fissure sealant manufactured by Arkona, Helioseal F, Helioseal F Plus, and Conseal. Tested parameters included emission of fluoride ions into saline (0.9% NaCl) and deionized water over a period of 2 weeks. Values were recorded after 1, 3, 24, 48, 72, and 96 h, and then after 1 and 2 weeks. All sealants were characterized by a constant fluoride release level. The highest fluoride release level was noted for Conseal (0.0169 ppm/mg), while the lowest was noted for fissure sealant manufactured by Arkona (0.063 ppm/mg). Fissure sealants, which contain fluoride, release it not only during polymerization, but also for several days after application. The use of fissure sealants whose composition includes fluoride is an effective method of preventing tooth decay.

## 1. Introduction

Tooth decay is one of the most common oral diseases. It occurs in all people, regardless of race, gender, or age, and is therefore classified as a lifestyle disease [1,2].

For years, it has been known that the best way to fight this disease is to prevent it; for this reason, dentistry employs a whole range of preventive procedures aimed at inhibiting the development of tooth decay. One of these is the use of the most cariostatic of elements, i.e., fluorine. Fluorine compounds have been playing a major role in tooth disease prevention for 70 years and are the most effective method of combating tooth decay [1].

Fluorine is one of the most common elements found in the environment. Due to its properties, it is widely used in medicine and dentistry as well as industry and agriculture.

As a highly bioactive element, it affects a number of processes in living organisms. Fluoride ions are inhibitors or, less frequently, activators of multiple enzymes; they affect protein biosynthesis as well as carbohydrate and lipid metabolism, which enables them to modify certain biological functions of living organisms [3,4,5,6].

Dentistry, however, makes use of their anticariogenic action—fluoride deposits itself in the enamel and reduces the production of acid by plaque bacteria, thus inhibiting demineralization and increasing remineralization [2,7].

Fluoride is being more and more frequently added to filling materials, including sealants; this provides a reserve of fluoride ions for hard dental tissues through gradual release over a longer period of time [8]. It has been proven that incorporation of fluoride into the composition of fissure sealing materials inhibits the initiation and progression of caries [9].

The aim of this study was to compare fluoride release from four selected fissure sealants—fissure sealant manufactured by Arkona, Helioseal F, Helioseal F Plus and Conseal—into two different media: deionized water and saline.

## 2. Materials and Methods

Four different fissure sealants were used in the study: Helioseal F (Ivoclar Vivadent), Helioseal F Plus (Ivoclar Vivadent), Conseal F (SDI), and fissure sealant manufactured by Arkona. Ten disc-shaped samples with a diameter of 4 mm and a height of 2 mm were made from each material. As per recommendations of the manufacturers, samples were cured using a Light Pro (GC) lamp for 20 s. The samples were then immersed in deionized water (5 samples of each material) and in NaCl solution (5 samples of each material) with a temperature of 37 °C.

An ORION 9609 ion-selective electrode, coupled with a CP-551 ELMETRON microcomputer pH/ion meter, calibrated before each measurement, was used to measure fluoride ion emission.

Fluoride release was checked after 1, 3, 24, 48, 72, and 96 h, and then after 1 and 2 weeks.

X-ray Diffraction (XRD) measurements were made on the X’Pert PRO X-ray diffractometer (Cu Kα1, 1.54060 Å) by PANalytical. Scanning electron microscope (SEM) micrographs were made on FEI Nova NanoSEM 230 microscope. Fourier Transform Infrared (FTIR) spectra measurements were performed on a Thermo Scientific Nicolet iS50 FTIR spectrometer equipped with an ATR module (iS50 ATR). The source of infrared radiation was a HeNe laser.

Descriptive statistics, including mean standard deviation (±SD) and 95% confidence interval (±95% CI), were calculated for each of the four studied groups. Data distribution was performed using the Shapiro–Wilk test of normality. ANOVA for dependent samples was used to determine if there were significant differences in fluoride ion release for each material during the fluoride ion release periods. ANOVA for independent groups was used to calculate the differences between the studied groups. A post-hoc Tukey’s test was performed to compare the studied groups. *p*-Values of < 0.05 were deemed statistically significant. Statistical analyses were performed using Statistica v.13.3 (Tibco Software Inc., Palo Alto, CA, USA).

## 3. Results

**Figure 1:**
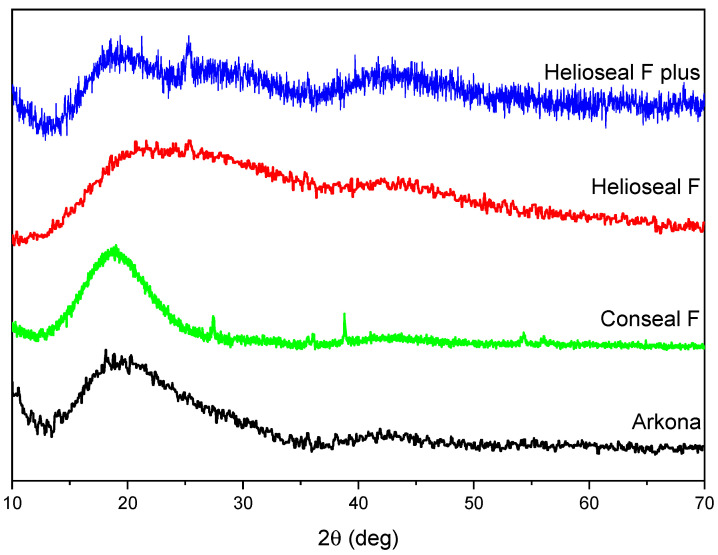
XRD diagrams of different fissure sealants. The study was performed for one randomly selected sample of each material: Arkona; Conseal F; Helioseal F; Helioseal F Plus.

Figure 1 shows the XRD patterns of different fissure sealants prepared in the form of pellets. In general, all sealants are amorphous, which is demonstrated by a broad diffraction peak centered at 2θ ≈ 20° for standard fissure sealants, i.e., Conseal F and Helioseal F plus, and at 2θ ≈ 25° for Helioseal F. It is worth noting that, for Conseal F, there are also some sharp peaks visible, which can be attributed to NaF and YbF_3_ crystalline phases. Their origin is from 7% submicron admixture, from which this material is made.

**Figure 2:**
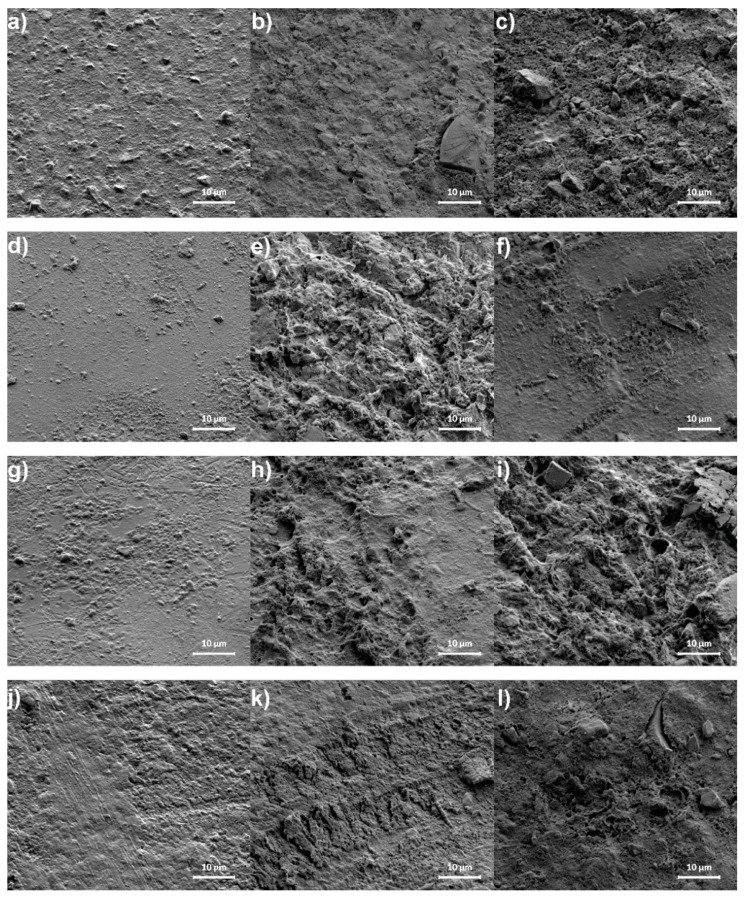
SEM micrographs of Arkona (**a**–**c**), Helioseal F (**d**–**f**), Conseal (**g**–**i**) and Helioseal F Plus (**j**–**l**) before, after release in deionized water and after release in saline solution, respectively. The study was performed for one randomly selected sample of each material: Arkona; Conseal F; Helioseal F; Helioseal F Plus.

SEM images of four different kinds of fissure sealants are presented in Figure 2. For each of these materials, an image was taken before and after release in deionized water and saline solution. All materials before release show quite a smooth surface without any visible crystalline fractions. This is probably caused by the fact that the NaF and YbF_3_ phases identified by XRD measurements are too small to be visible in this magnification. After release of fluoride ions, the surface of each sample become more porous and, for the Conseal sample, deep channels are visible both for release into deionized water and saline solution. Moreover, some crystalline structures (probably NaCl crystals) are visible for all samples after release in saline solution.

**Figure 3:**
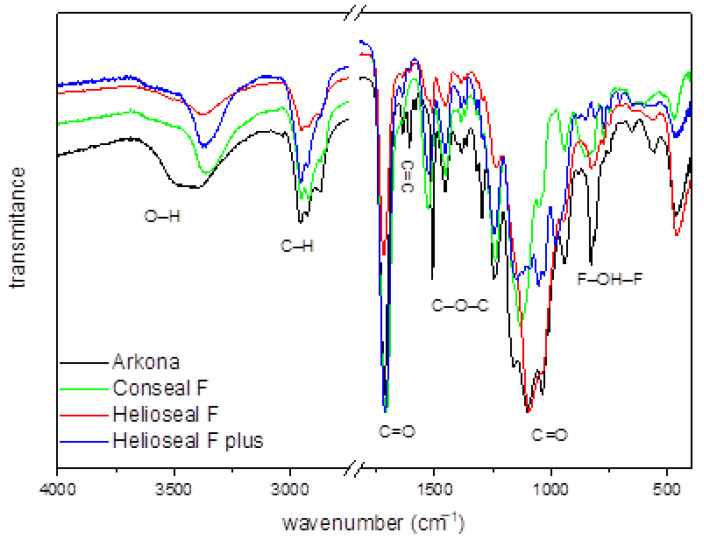
FTIR spectra of different fissure sealant materials. The study was performed for one randomly selected sample of each material: Arkona; Conseal F; Helioseal F; Helioseal F Plus.

Figure 3 presents the FTIR spectrum with assigned characteristic bands for the organic sealant materials. The bands from region of 1000 to 1200 and 1712 cm^−1^ originate from the vibrations of the C=O double bond, the band at 1250 cm^−1^ is related to the vibration of the C–O–C molecule, the band located at 1610 cm^−1^ corresponds to the characteristic for dental materials C=C double bond, while intense bands at 2866 and 2960 cm^−1^ are related to the vibrations of the C-H bond. Since all materials contain fluoride, there is also a visible peak at 749.7 cm^−1^ for the F–OH–F configuration. Moreover, the OH− group could be substituted in two positions (F–OH–F or F–HO–F) and might be located from the one to the another with relatively low energy [10]. A small difference in the location between particular peaks is noticeable, which could be related with fluoride ions into the fissure sealant materials. In addition, a weak band is visible around 3500 cm^−1^ associated with the vibration of the O–H group.

**Figure 4:**
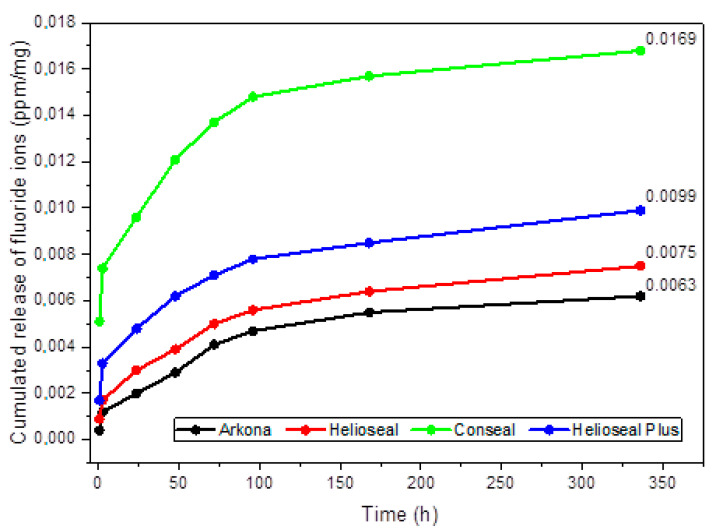
Cumulative fluoride ion release (ppm/mg) from fissure sealants into 0.9% NaCl. Dots represent the means of measurements.

Emission of fluoride ions from the studied fissure sealants manufactured by Arkona, Helioseal, Conseal and Helioseal Plus—into a saline solution is shown in Table 1. Statistically significant differences in fluoride release over the given time periods were found for all materials (*p* = 0.0005, *p* < 0.0001 and *p* = 0.0034). Arkona exhibited the highest fluoride release level at 72 h of incubation, Helioseal at 24 h of incubation, Conseal at 1 h of incubation, and Helioseal Plus at between 1 and 3 h of incubation. As shown in Figure 4, the highest cumulative fluoride release level was noted for the Conseal material (0.0169 ppm/mg), with the second-highest noted for Helioseal Plus (0.099 ppm/mg), the third-highest for Helioseal (0.075 ppm/mg), and the lowest for the fissure sealant manufactured by Arkona (0.063 ppm/mg).

Table 1 shows the mean fluoride release levels. The highest mean fluoride release level was observed for Conseal (0.0021 ± 0.0015 ppm/mg) and Helioseal plus (0.0012 ± 0.0006 ppm/mg). The lowest mean fluoride release level was noted for the fissure sealant manufactured by Arkona and for Helioseal (0.0008 ± 0.0003 ppm/mg and 0.0009 ± 0.0003 ppm/mg, respectively). There were statistically significant differences in fluoride ion release between the fissure sealant manufactured by Arkona and Conseal, between Helioseal and Conseal and between Helioseal Plus and Conseal (*p* < 0.0001 for all).

**Figure 5:**
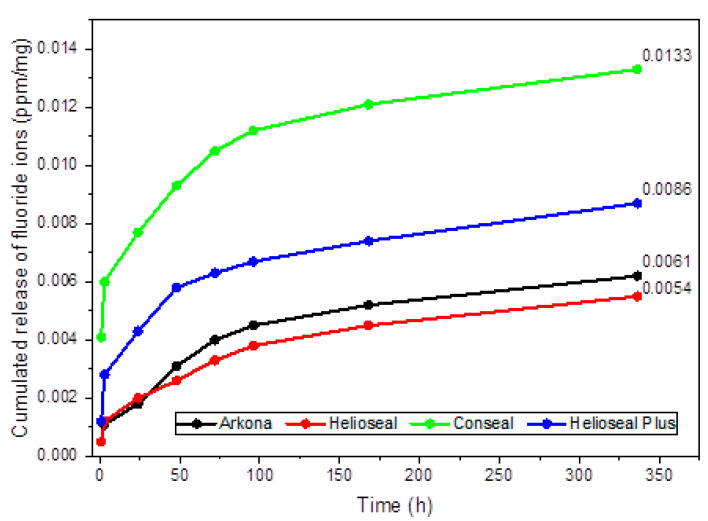
Cumulative fluoride ion release (ppm/mg) from fissure sealants into deionized water. Dots represent the means of measurements.

Table 2 shows the results for fluoride ion release from the studied fissure sealants into deionized water. There were statistically significant differences in fluoride emission between periods: fissure sealant manufactured by Arkona (*p* < 0.0004), Conseal (*p* < 0.0001), and Helioseal Plus (*p* = 0.0001). The highest fluoride ion release for Arkona was recorded at 48 h of incubation, for Helioseal at 2 weeks of incubation, for Conseal at 1 h of incubation, and for Helioseal Plus at 3 h of incubation. Figure 5 shows the release of cumulative fluoride ion into deionized water. The highest cumulative fluoride release level was noted for Conseal (0.0133 ppm/mg), followed by Helioseal Plus (0.086 ppm/mg), the fissure sealant manufactured by Arkona (0.061 ppm/mg), and, finally, Helioseal (0.054 ppm/mg).

There were significant differences between the mean fluoride release levels from the fissure sealant manufactured by Arkona, Helioseal, Conseal, and Helioseal Plus (Table 2). Significant differences were noted between the fissure sealant manufactured by Arkona and Conseal, between Helioseal and Conseal and between Helioseal Plus and Conseal (*p* < 0.001 for all).

**Figure 6:**
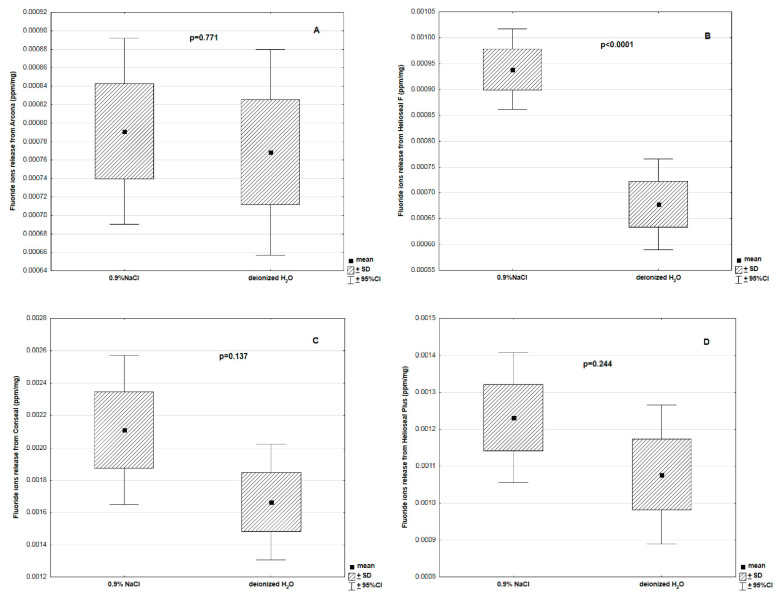
Fluoride ion release from different fissure sealants ((**A**)—Arkona; (**B**)—Helioseal; (**C**)—Conseal; (**D**)—Helioseal Plus) into 0.9% NaCl and deionized water in a period of 336 h.

Figure 6 shows comparative analyses of mean fluoride release levels into a saline solution and deionized water for each tested material. Only Helioseal exhibited a significantly higher fluoride emission level into saline solution compared to deionized water (*p* < 0.0001).

## 4. Discussion

Systematic preventive procedures performed at the dentist’s office are an important part of the fight against tooth decay. They are also the basic element of difficult interdisciplinary dental treatment, especially in children with disabilities as well as in patients with systemic diseases [11,12,13,14]. One such procedure is the sealing of anatomical fissures and pits of permanent molars right after eruption using fissure sealants.

Sealants are liquid fillings which are not permanent, but effectively protect the tooth by releasing fluoride. As early as the 1930s, fluoride was shown to significantly reduce the severity of caries [15].

Fluorine is a cyclical element that is very common in nature. It is the thirteenth most abundant element in the Earth’s crust and is a constituent of multiple minerals, e.g., fluorite, cryolite, fluorapatite, and topaz.

Fluorine is also classified as a trace element and is present in mammals at 500 mg/kg of dry mass [16].

Daily fluoride intake requirements of the human body vary with age. According to Moody [17], fluoride intake should not exceed an average of 1.2 mg/day for children, 4.2 mg/day for adult males, and 3.6 mg/day for adult females. On the other hand, Olczak-Kowalczyk et al. [18,19] specified daily fluoride intake requirements at between 0.01 mg for infants and 3.0 mg for adults (Table 3).

Fluorine is one of the most electronegative elements and, in ionized form, has a strong affinity for hydroxyl ion (OH^−^) exchange in hydroxyapatite. Electrostatically, the interaction of calcium and fluoride (F^−^) ions is greater than that of Ca^2+^ and OH^−^ ions, which ensures higher stability of the crystal lattice and, consequently, lower solubility of apatite in acids. These data provide an explanation for the origin of the long-standing principle of fluoride action, which boils down to “fluorides make teeth more resistant to caries” [18].

The source of fluoride in sealants is fluorosilicate glass. According to modern guidelines of scientific societies related to prevention of tooth decay, long-term fluoride release in low concentrations is more effective than release of high doses in a short period of time [18].

The ability to prevent caries development as a result of low release of fluoride ions from dental materials has not been fully explained. According to Dijkman and Arends [20], low fluoride emission from materials is unfavorable, as the optimal concentration for preventing caries development at the tooth/filling interphase should be between 5 and 80 ppm. On the other hand, Featherstone [21] postulated that significant remineralization occurs even at very low fluoride ion concentrations of 0.03–0.05 ppm.

Sadrabad et al. [22] observed that fluoride released from sealants is not only effective in inhibiting tooth decay, but its remineralization capacity exceeds that of fluoride released from toothpaste.

Cagetti et al. [23], in turn, demonstrated that fluoride-containing sealants provide protection against caries on the distal surface of second molars. The antibacterial and remineralizing effect of fluoride contained in fissure sealants contributes to the success of new therapies, such as the use of stem cells in bone and dental loss defects regeneration [24,25,26].

The dynamics of fluoride ion release are affected by many factors, e.g., concentration of fluoride ions in the preparation and additional active compounds which interact with fluoride [27,28,29].

Williams et al. [30] proved that fluoride release is dependent on the surface area of the material.

In our study, the choice of contact fluids in which the samples were immersed was dictated by the fact that deionized water eliminates the interactions between fluoride ions and other ions, while saline is an electrolyte that is similar to human saliva.

Our study shows that as many as three out of four tested fissure sealants (Conseal, Helioseal F, Helioseal F Plus) exhibit a higher degree of fluoride ion emission into saline, although the difference was statistically significant only for Helioseal F. The fissure sealant manufactured by Arkona released the same number of fluoride ions into both media.

Results of our study are, therefore, inconsistent with results obtained by most authors. Most studies indicate that fluoride release is greater in deionized water than in artificial and human saliva or a saline solution [31,32,33,34,35,36,37]. This phenomenon is explained by a lower diffusion gradient between filling materials and the ion-enriched human saliva or saline solution compared to the difference in gradients between filling materials and deionized water [38].

In their study of materials, Rezk-Lega et al. [39] and Mallakh et al. [40] found that fluoride release is greater in deionized water than in a saline solution.

Testing indicated statistically significant differences in fluoride release over the given time periods for all materials in each medium.

For the Conseal sealant, the highest fluoride release level was noted after only one hour of incubation, both in deionized water and in saline, while, for the Helioseal F Plus sealant, the highest level was observed after 3 h for both media.

The other two sealants exhibited significant differences in the timing of the highest ion release level.

For Helioseal F, the highest fluoride emission was noted at 24 h of incubation in saline, compared to 2 weeks for deionized water, while the fissure sealant manufactured by Arkona showed the highest emission at 72 h of incubation in saline and 48 h of incubation in deionized water.

These results are consistent with results obtained by other researchers [41,42,43,44,45].

When assessing fluoride release from giomers and fissure-sealing resins, Sismanoglu [45] noted that sealants characterized by high and long-term fluoride release should be the primary choice for patients at high risk of caries. In a long-term study using the FluroShield fissure sealant, Rock et al. [46] showed that the material exhibited a low but stable fluoride release for as long as 6 months.

## 5. Conclusions

All prepared samples have polymerized properly, which was confirmed by existence of amorphic phases in the XRD diagram and in typical bands for dental materials in the FT-IR spectra.

Fissure sealants, which contain fluoride, release it not only during polymerization, but also for several days after application. Further studies are needed to determine the long-term capacity of sealants to release fluoride.

Each of the fissure sealants released the largest number of fluoride ions in a different time interval. The highest mean fluoride release level was observed for Conseal sealant after placing samples into saline solution as well as into deionized water. The above material also showed the highest cumulative fluoride ion release into the both solutions.

The use of fissure sealants whose composition includes fluoride is an effective method of preventing tooth decay.

## Figures and Tables

**Table 1:** Fluoride ion release (ppm/mg) from fissure sealants into a saline solution (0.9% NaCl). Descriptive data are presented as mean values + standard deviation (SD).

Time	Arkona (ppm/mg)	Helioseal (ppm/mg)	Conseal (ppm/mg)	Helioseal Plus (ppm/mg)
1 h	0.0004 ± 0.0001	0.0009 ± 0.0001	0.0051 ± 0.0018	0.0017 ± 0.0007
3 h	0.0008 ± 0.0003	0.0008 ± 0.0001	0.0023 ± 0.0006	0.0016 ± 0.0004
24 h	0.0008 ± 0.0001	0.0013 ± 0.0003	0.0022 ± 0.0003	0.0015 ± 0.0004
48 h	0.0009 ± 0.0002	0.0009 ± 0.0001	0.0025 ± 0.0010	0.0014 ± 0.0003
72 h	0.0012 ± 0.0002	0.0011 ± 0.0001	0.0016 ± 0.0006	0.0009 ± 0.0006
96 h	0.0006 ± 0.0001	0.0006 ± 0.0001	0.0011 ± 0.0006	0.0007 ± 0.0002
1 week	0.0008 ± 0.0002	0.0008 ± 0.0001	0.0009 ± 0.0002	0.0007 ± 0.0002
2 weeks	0.0007 ± 0.0005	0.0011 ± 0.0002	0.0011 ± 0.0005	0.0014 ± 0.0006
Mean value ± SD	0.0008 ± 0.0003	0.0009 ± 0.0003	0.0021 ± 0.0015	0.0012 ± 0.0006
*p*-value (ANOVA for dependent samples)	0.0005 *	<0.0001 *	<0.0001 *	0.0034 *
*p*-value (ANOVA for independent groups)	<0.0001 *			
Tukey’s post-hoc test:				
Arkona	-			
Helioseal	0.854	-		
Conseal	<0.0001 *	<0.0001 *	-	
Helioseal Plus	0.079	0.385	<0.0001 *	-

* statistically significant.

**Table 2:** Fluoride ion release (ppm/mg) from fissure sealants into deionized water. Descriptive data are presented as mean values + standard deviation (SD).

Time	Arkona (ppm/mg)	Helioseal (ppm/mg)	Conseal (ppm/mg)	Helioseal Plus (ppm/mg)
1 h	0.0005 ± 0.0002	0.0005 ± 0.0001	0.0041 ± 0.0014	0.0012 ± 0.0002
3 h	0.0006 ± 0.0002	0.0007 ± 0.0004	0.0019 ± 0.0007	0.0016 ± 0.0009
24 h	0.0007 ± 0.0001	0.0008 ± 0.0004	0.0017 ± 0.0002	0.0015 ± 0.0005
48 h	0.0013 ± 0.0004	0.0006 ± 0.0002	0.0016 ± 0.0003	0.0015 ± 0.0005
72 h	0.0009 ± 0.0002	0.0007 ± 0.0002	0.0012 ± 0.0003	0.0005 ± 0.0001
96 h	0.0005 ± 0.0001	0.0005 ± 0.0001	0.0007 ± 0.0002	0.0004 ± 0.0001
1 week	0.0007 ± 0.0002	0.0007 ± 0.0001	0.0009 ± 0.0001	0.0007 ± 0.0001
2 weeks	0.0010 ± 0.0005	0.0010 ± 0.0003	0.0012 ± 0.0005	0.0013 ± 0.0004
Mean value ± SD	0.0008 ± 0.0004	0.0007 ± 0.0003	0.0017 ± 0.0012	0.0011 ± 0.0006
*p*-value (ANOVA for dependent samples)	0.0004 *	0.143	<0.0001 *	0.0001 *
*p*-value (ANOVA for independent groups)	<0.0001 *			
Tukey’s post-hoc test:				
Arkona	-			
Helioseal	0.936	-		
Conseal	<0.0001 *	<0.0001 *	-	
Helioseal Plus	0.190	0.049 *	0.0009 *	-

* statistically significant.

**Table 3:** Daily fluoride intake requirements of the human body: optimal and acceptable.

Age	Daily Fluoride Intake Requirements of the Human Body in mg/day	Upper Acceptable Limit of Fluoride Intake in mg/day
0–6 months	0.01	0.7
6–12 months	0.5	0.9
1–3 years	0.7	1.3
4–8 years	1.0	2.2
9–13 years	2.0	2.8
14–18 years	3.0	3.6

## Data Availability

Not applicable.
